# Retinal and optic nerve changes in microcephaly: An optical coherence tomography study

**DOI:** 10.1212/WNL.0000000000005950

**Published:** 2018-08-07

**Authors:** Eleni Papageorgiou, Anastasia Pilat, Frank Proudlock, Helena Lee, Ravi Purohit, Viral Sheth, Pradeep Vasudevan, Irene Gottlob

**Affiliations:** From the Department of Ophthalmology (E.P., A.P., F.P., H.L., R.P., V.S., I.G.), Leicester Royal Infirmary, University of Leicester; and Department of Clinical Genetics (P.V.), University Hospitals of Leicester, Leicester Royal Infirmary, UK.

## Abstract

**Objective:**

To investigate the morphology of the retina and optic nerve (ON) in microcephaly.

**Methods:**

This was a prospective case-control study including 27 patients with microcephaly and 27 healthy controls. All participants underwent ophthalmologic examination and handheld optical coherence tomography (OCT) of the macula and ON head. The thickness of individual retinal layers was quantified at the foveal center and the parafovea (1,000 μm nasal and temporal to the fovea). For the ON head, disc diameter, cup diameter, cup-to-disc ratio, cup depth, horizontal rim diameter, rim area, peripapillary retinal thickness, and retinal nerve fiber layer thickness were measured.

**Results:**

Seventy-eight percent of patients had ophthalmologic abnormalities, mainly nystagmus (56%) and strabismus (52%). OCT abnormalities were found in 85% of patients. OCT revealed disruption of the ellipsoid zone, persistent inner retinal layers, and irregular foveal pits. Parafoveal retinal thickness was significantly reduced in patients with microcephaly compared to controls, nasally (307 ± 44 vs 342 ± 19 μm, *p* = 0.001) and temporally (279 ± 56 vs 325 ± 16 μm, *p* < 0.001). There was thinning of the ganglion cell layer and the inner segments of the photoreceptors in microcephaly. Total peripapillary retinal thickness was smaller in patients with microcephaly compared to controls for both temporal (275 vs 318 μm, *p* < 0.001) and nasal sides (239 vs 268 μm, *p* = 0.013).

**Conclusions:**

Retinal and ON anomalies in microcephaly likely reflect retinal cell reduction and lamination alteration due to impaired neurogenic mitosis. OCT allows diagnosis and quantification of retinal and ON changes in microcephaly even if they are not detected on ophthalmoscopy.

Ocular abnormalities in microcephaly include chorioretinal degeneration, pigmentary changes, optic disc coloboma and atrophy, falciform retinal folds, microphthalmia, hypoplastic fovea, strabismus, and nystagmus.^1–10^ It is hypothesized that the neural and ocular anomalies in microcephaly are due to the linked development of the eye and the brain, as the retina represents an outpocketing of the neuroepithelium.^11^ However, the extent to which the retina and optic nerve are affected in microcephaly is unknown. Recently, optical coherence tomography (OCT) in 4 patients with *KIF11* mutations and microcephaly with or without chorioretinopathy, lymphedema, or intellectual disability revealed atrophic maculae and disruption of the photoreceptor inner segment ellipsoid and the outer retinal bands.^5^ Foveal hypoplasia, retinal pigment epithelium (RPE) thinning, and a disruption of the ellipsoid zone (EZ) also have been found.^12^ In addition, children with congenital Zika syndrome showed retinal and choroidal thinning.^13^

The case series were descriptive, and to date no objective comparison of foveal and optic disc morphology between patients with microcephaly and age-matched controls has been performed. Obtaining OCTs in microcephaly even in neonates has recently been made possible due to the advent of handheld OCT (HH-OCT), which is suitable for patients who do not cooperate well.^12^

In this study, we compared the individual retinal layers and optic disc measures between children with microcephaly and healthy children by using HH-OCT. We hypothesized that children with microcephaly have thinner retinae and smaller optic discs compared to healthy participants. We describe the ocular phenotype in 27 children with microcephaly, representing the largest series to date to undergo detailed structural retinal assessment by using HH-OCT.

## Methods

### Participants

Twenty-seven patients with primary microcephaly (15 female, 12 male, mean age 9.4 ± 7.8 years) and 27 age-matched healthy controls (15 female, 12 male, mean age 9.0 ± 7.2 years) were included in this prospective observational study. Children with microcephaly were recruited from the Department of Ophthalmology and the Department of Clinical Genetics at the University Hospitals of Leicester, UK. For each patient, we compared one normal age-, sex-, and ethnicity-matched healthy control. Control participants were identified from the Leicestershire region within the National Health Service during routine pediatric outpatient clinic appointments. Control participants were excluded if they had any preexisting ocular, neurologic, or metabolic abnormalities. All participants underwent a standard ophthalmologic examination, including best-corrected visual acuity (where possible), subjective or objective cycloplegic refraction, orthoptic examination, slit-lamp examination, and funduscopy. In younger infants and children unable to cooperate with optotype visual acuity, visual acuity was assessed by preferential looking using Teller acuity cards. In cooperative participants, we used uncrowded or if possible crowded optotypes (Kay pictures) or Snellen test chart. The medical records of all 27 patients were reviewed for perinatal history, family history, growth measures, presence of dysmorphic features, neurodevelopmental status, neuroimaging studies, and chromosome studies. The presence of microcephaly was established by a clinical geneticist on the basis of decreased head circumference on clinical examination. Twelve out of 27 individuals had a likely syndromic form of microcephaly. All affected individuals had a standard karyotyping (G banding) or microarray investigation using the ISCA 8 × 60 k v2 array (Agilent, Santa Clara, CA; or BlueGnome, Cambridge, UK). Neuroimaging (MRI or CT) had been obtained in 22 out of 27 patients.

### Standard protocol approvals, registrations, and patient consents

Ethics committee approval was granted and the research study was performed according to the Declaration of Helsinki. Following verbal and written explanation of the experimental protocol, all participants or parents/guardians of participants gave their written consent.

### Handheld optical coherence tomography

The retinae and optic discs of the study participants were scanned with a portable, noncontact, HH spectral-domain OCT device (Leica Microsystems, Buffalo Grove, IL) using a protocol described previously.^14^ All children were scanned in an outpatient clinic setting without sedation. In some cases, the pupils were dilated with cyclopentolate 1%. Where possible, both eyes were scanned. All tests were conducted on the same day by 3 different examiners (E.P., R.P., V.S.) in the same examination room.

We were successful in obtaining scans on one or both eyes in all participants on the day of examination. In 100% of controls and 90% of patients, the scans were successfully obtained without mydriasis. Successful HH-OCT scans took between 2 and 5 minutes in cooperative children and up to 30 minutes in less cooperative children. Scanning of younger infants was most effective when the child was seated on the parent's lap while bottle feeding or breastfeeding. For older children, scanning was performed while watching age-appropriate cartoons using a portable laptop computer.

A 3D raster scan program consisting of 100 B-scans and 500 A-scans per B-scan line was used to capture the foveal, parafoveal, and optic disc regions. The scanning window covered a 10 × 5-mm retinal area centered on the fovea and a 5 × 5-mm optic nerve head (ONH) area centered on the optic disc excavation. The acquisition time for each volumetric scan was 2.9 seconds, which minimizes any motion artefacts caused by nystagmus. The acquired images were exported from the Leica HH spectral-domain–OCT software and imported into ImageJ software (available at rsbweb.nih.gov/ij/). All OCT images fulfilled the OSCAR-IB quality control criteria for retinal OCT scans.^15^ In cases where OCT scans had been acquired from both eyes, the scan with the best image quality was included in the analysis. In all participants for whom scans were available for both eyes, there was no intereye difference in the configuration of the foveal and the optic nerve head tomograms. The 9-point advised protocol for OCT study terminology and elements (Advised Protocol for OCT Study Terminology and Elements [APOSTEL] recommendations) is presented in table e-1 (links.lww.com/WNL/A617).^16^

### Foveal analysis

**Figure 1:**
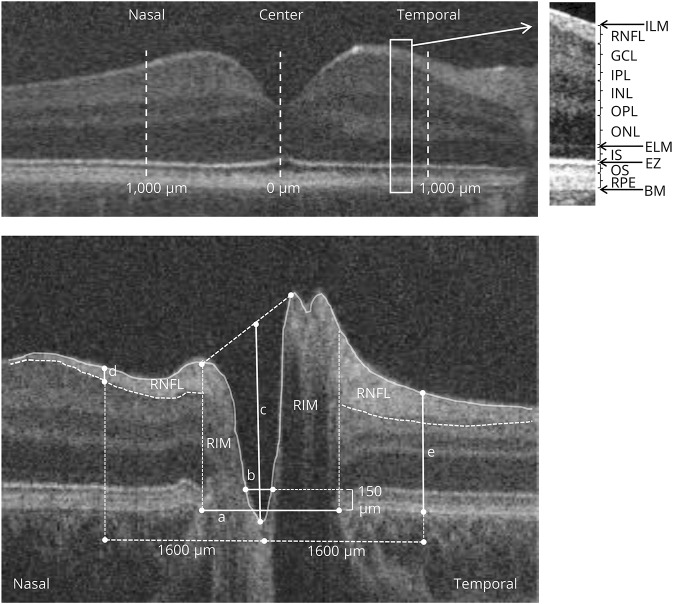
Horizontal spectral-domain optical coherence tomography (OCT) images Images of the (A) macula and (B) optic nerve head of a control participant. (A) The position of the different retinal layers (right) in an OCT scan of the macular area (left). The thickness of the layers was measured at the foveal center and the parafovea (1,000 μm temporally and 1,000 μm nasally from the fovea). (B) The disc diameter (a) was defined as the distance between the edges of retinal pigment epithelium (RPE); cup diameter (b) was measured as the length of the line at 150 μm anterior to the disc plane; cup depth (c) was defined as the vertical distance from cup base to midpoint of neuroretinal peaks; horizontal rim diameter is the difference between horizontal disc and cup diameters (nasal and temporal rim were calculated as the difference between the disc and cup edges). Retinal nerve fiber layer (RNFL) thickness (d) and peripapillary retinal thickness (e) were measured at 1,600 μm on both sides of the center of the cup. RNFL was delineated manually (hyperreflective tissue inferior to the internal limiting membrane [ILM]), and peripapillary retinal thickness was measured from ILM to Bruch membrane (BM). EZ = ellipsoid zone; ELM = external limiting membrane; GCL = ganglion cell layer; INL = inner nuclear layer; IPL = inner plexiform layer; IS = inner segment; ONL = outer nuclear layer; OPL = outer plexiform layer; OS = outer segment.

The fovea was identified on the basis of visual inspection of the scans for its characteristic features, which include the deepest point of the foveal pit, thinning of the inner retinal layers, doming of the ONL, and lengthening of the photoreceptor outer segments.^17^ Retinal layer segmentation was performed in a semiautomated manner, using an ImageJ macro with the retinal layer borders positioned manually by locating points that were fitted with a spline fit.^18^ The thickness of individual foveal layers was quantified and compared between patients and healthy controls (retinal nerve fiber layer [RNFL], ganglion cell layer [GCL], inner plexiform layer [IPL], inner nuclear layer [INL], outer plexiform layer [OPL], outer nuclear layer [ONL], inner segments of the photoreceptors [IS], outer segments of the photoreceptors [OS], RPE). Measurements were performed at the foveal center and 1,000 μm nasally and temporally from the fovea. In addition, the following measures were calculated: total retinal thickness, thickness of inner layers consisting of the RNFL, GCL, IPL, INL, and OPL, and thickness of outer layers consisting of the ONL, IS, OS, RPE (figure 1A).

### ONH analysis

Quantitative OCT analysis was conducted in a semi-automated manner on a flattened B-scan through the deepest point of the optic nerve cup by using an ImageJ macro. The edges of the RPE, the position of the internal limiting membrane, and the RNFL position were marked manually, as described previously.^18,19^ The disc diameter (distance between the edges of the RPE), cup diameter (length of the line parallel to the disc diameter at 150 μm anteriorly to the disc diameter and limited by the internal limiting membrane), cup depth (vertical distance from cup base to midpoint of neuroretinal peaks), and horizontal rim diameter (difference between horizontal disc and cup diameters) were measured automatically by a macro (figure 1B). Peripapillary retinal thickness and RNFL thickness were measured at 1,600 μm at both sides of the center of the cup, as described previously.^18,19^

### Statistical analysis

All analyses were considered significant at a type 1 probability value of *p* < 0.05. Statistical analysis was performed with SPSS software version 24.0 (SPSS, Inc., Chicago, IL). The distribution of the optic nerve head and macular measurements were tested for normality using the Shapiro-Wilk test. For each retinal and ONH parameter, differences between the microcephaly group and controls were tested using general linear models that included group as a fixed factor with age and spherical equivalent included as covariates to adjust for age and refractive error, respectively.

For calculating correlations of visual acuity with OCT measures, the visual acuity fix and follow was converted to 2.28 logMAR visual acuity.^14^ Patients 22 and 23, who did not respond to light stimuli, were excluded from this analysis.

Reproducibility and reliability analysis of foveal and optic nerve measurements using similar methods to the present study have been reported previously.^14,19^ For foveal analysis, in a cohort of children from birth to 7 years of age, there was excellent degree of reproducibility between 2 examiners (intraclass correlation coefficient [ICC] >0.96 for central macular thickness and ICCs >0.8 for the outer nuclear layer and outer segment of the photoreceptors). The nerve fiber layer, ganglion cell layer, outer plexiform layer, inner segment of the photoreceptors, and RPE were less reliable, with ICCs <0.7. For optic nerve analysis, interexaminer reliability in children from birth up to 13 years of age was excellent for diametric and retinal thickness measures (ICC >0.89).

### Data availability

Anonymized data not published within this article will be made available by request from any qualified investigator.

## Results

The clinical details of the 27 patients are summarized in the table. A description of a number of selected cases follows below.

**Table:**
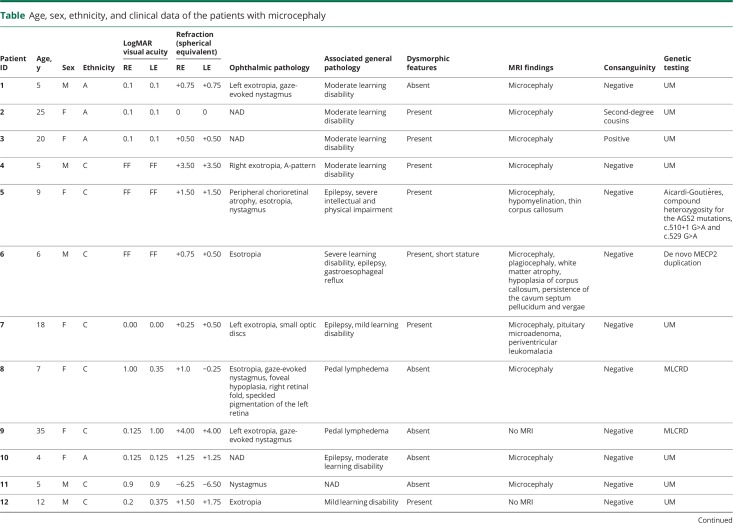

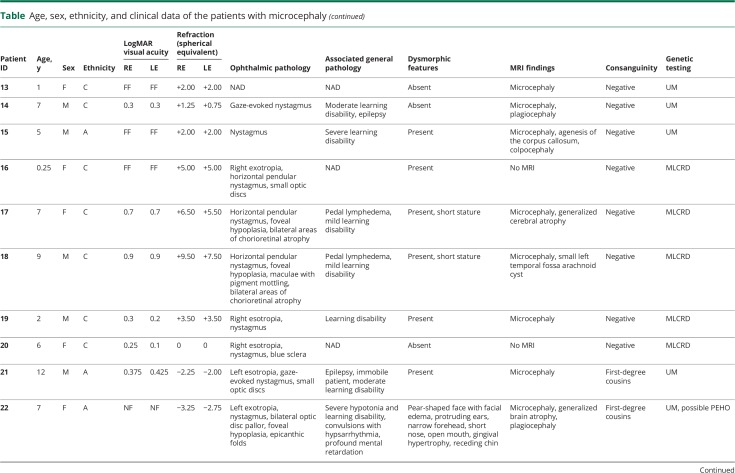

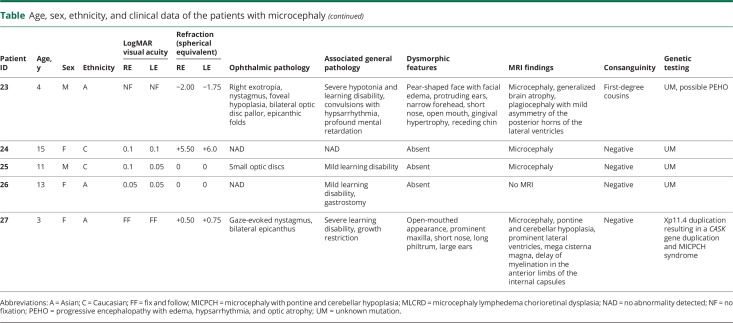
Age, sex, ethnicity, and clinical data of the patients with microcephaly

### Selected cases

#### Microcephaly lymphedema chorioretinal dysplasia (family 1)

**Figure 2:**
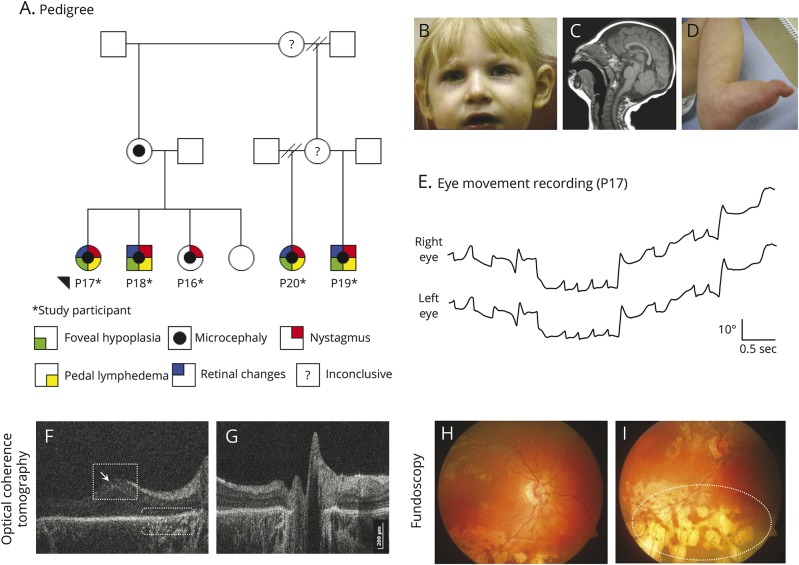
Family with microcephaly lymphedema chorioretinal dysplasia (A) Family tree. (B–I) Clinical signs of patient 17. (B) Facial features include broad nose with rounded tip, long philtrum with thin upper lip, and prominent ears. (C) MRI shows an anatomically normal but microcephalic brain. (D) Lymphedema of the feet. (E) Horizontal eye movement recordings demonstrate horizontal jerk nystagmus with increasing slow phase velocities (upward deflections indicate eye movements to the right, a downward deflection indicates eye movements to the left). (F) Macular handheld (HH) optical coherence tomography (OCT) displays foveal hypoplasia and mild macular elevation (open square). There is thinning of the retinal pigment epithelium (RPE) and hyperreflectivity underlying the RPE (brackets), and there are abnormal hyperreflective interfaces between the retinal nerve fiber layer and ganglion cell layer (arrow). (G) Normal optic nerve on HH-OCT. (H) In the posterior pole, there is mild retinal vascular tortuosity. (I) Peripheral chorioretinal atrophic lacunae (white oval).

Three siblings (patients 16, 17, and 18) were diagnosed with microcephaly lymphedema chorioretinal dysplasia (MLCRD) due to microcephaly, restricted growth, pedal lymphedema, horizontal jerk nystagmus, chorioretinal atrophic changes, and significant hypermetropia (figure 2). Mild dysmorphic features including broad nose with rounded tip, pointed chin, and prominent ears were noted. Both older siblings had reduced visual acuity and foveal hypoplasia on HH-OCT (figure 2F). The younger sibling (patient 16) had optic nerve hypoplasia. The first cousins (patients 19 and 20) of the above siblings also presented with microcephaly, nystagmus, and strabismus. Genetic testing of this family did not show a mutation in the *KIF11* gene.

#### Microcephaly with pontocerebellar hypoplasia (Xp11.4 duplication, *CASK* duplication)

**Figure 3:**
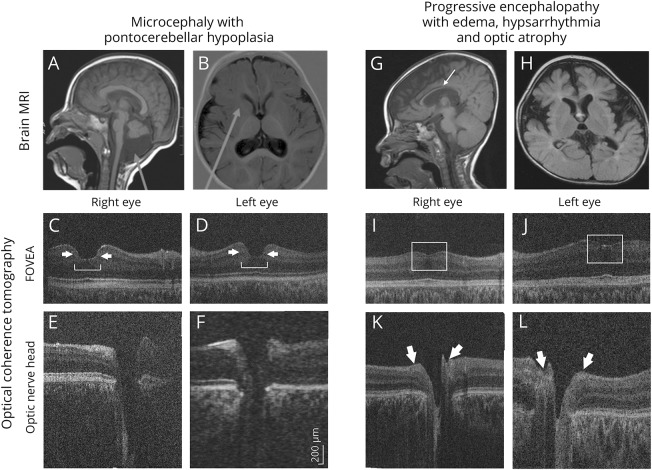
Patient 27 with microcephaly with pontine and cerebellar hypoplasia (MICPCH) and patient 22 with possible progressive encephalopathy with edema, hypsarrhythmia, and optic atrophy (PEHO) (A, B) MRI brain of patient 27 with MICPCH shows pontocerebellar hypoplasia, mega cisterna magna (A, arrow), and delayed myelination at the anterior limbs of the internal capsule (B, arrow). (C, D) Macular handheld (HH) optical coherence tomography (OCT) of the right and left eye displays a broadened fovea (bracket) with steep borders (arrows). (E, F) Optic nerve HH-OCT of the right and left eye with an enlarged cup-to-disc ratio of 0.8 at both eyes. (G, H) MRI brain of patient 22 with possible PEHO shows generalized brain atrophy, plagiocephaly, thin corpus callosum (arrow), and retarded myelination. (I, J) Macular HH-OCT of the right and left eye of patient 22 demonstrates diminished foveal depression due to foveal hypoplasia (open squares). (K, L) Optic nerve HH-OCT of the right and left eye of patient 22 with thinning of the peripapillary retinal nerve fiber layer (arrows).

A 3-year-old Asian girl (patient 27) with severe learning difficulties and growth restriction had been diagnosed with microcephaly, pontine, and cerebellar hypoplasia (MICPCH) syndrome (figure 3, A and B). Family history was unremarkable. Clinical examination revealed microcephaly, gaze-evoked nystagmus, growth restriction, developmental delay, and dysmorphic facial features. In HH-OCT, the fovea was broadened, and the foveal border was steep (figure 3, C and D). Array comparative genomic hybridization analysis revealed *CASK* gene duplication at Xp11.4.

#### Progressive encephalopathy with edema, hypsarrhythmia, and optic atrophy syndrome

Two siblings (patients 22 and 23) with consanguineous parents had infantile spasms with hypsarrhythmia, profound mental retardation, severe arrest of motor development, and subcutaneous edema of the face, hands, and feet. Visual fixation was absent. Both patients had microcephaly, dysmorphic features, exotropia, drooping puffy eyelids, nystagmus, and bilateral optic atrophy. HH-OCT revealed foveal hypoplasia (figure 3, I–L). MRI showed generalized brain atrophy, plagiocephaly, thin corpus callosum, and delayed myelination (figure 3, G and H). The clinical characteristics in combination with the neuroradiologic findings were compatible with progressive encephalopathy with edema, hypsarrhythmia, and optic atrophy (PEHO) syndrome.

#### Diagnosis and inheritance

Investigations including TORCH screening, urinary amino acids, and cytogenetics (karyotype/microarray) were normal in all patients. Diagnoses are listed in the table. All participants were born after 37 weeks gestation and pregnancy was uncomplicated. None of the patients had a history of intrauterine or perinatal teratogen exposure and none of the mothers of affected children had signs or symptoms of Zika virus infection or other infections during pregnancy. Seven patients (from 2 families) had a clinical diagnosis of MLCRD (*KIF11* negative). There were 2 siblings with possible PEHO syndrome, 1 patient with Aicardi-Goutières syndrome, 1 patient with de novo *MECP2* duplication, and 1 patient with MICPCH syndrome. In the remaining 15 patients, the microcephaly could not be classified further (unknown mutation, see the table). An autosomal recessive mode of inheritance was probable in 5 affected children of consanguineous parents (patients 2, 3, 21, 22, and 23) and 2 affected siblings of unrelated parents (patients 24 and 25).

#### Ophthalmologic abnormalities

Ophthalmologic abnormalities were present in 21 out of 27 (78%) patients. Best-corrected visual acuity ranged from 0.05 to 0.9 logMAR (from 6/60 to 6/5 Snellen in older patients, table). Seven patients were able to fix and follow; the 2 siblings with PEHO syndrome (patients 22 and 23) were not able to fix and follow and did not respond to light stimuli. Most patients had mild hypermetropia, except for 4 patients with myopia. Five patients had hypermetropia >+4 sphere and one patient had myopia >−4 sphere.

The most common ophthalmologic abnormalities were nystagmus (56%) and strabismus (52%). Gaze-evoked nystagmus was the most common nystagmus type (22%). The most common strabismus type was exotropia (30%), followed by esotropia (22%). Seven patients were diagnosed with amblyopia (26%). Optic nerve hypoplasia was found in 4 patients (15%). Chorioretinal dysplasia with punched-out lacunar lesions and chorioretinal atrophic areas occurred in 3 patients with MLCRD. Falciform retinal folds were seen in one patient with MLCRD (4%).

#### Macular morphology

**Figure 4:**
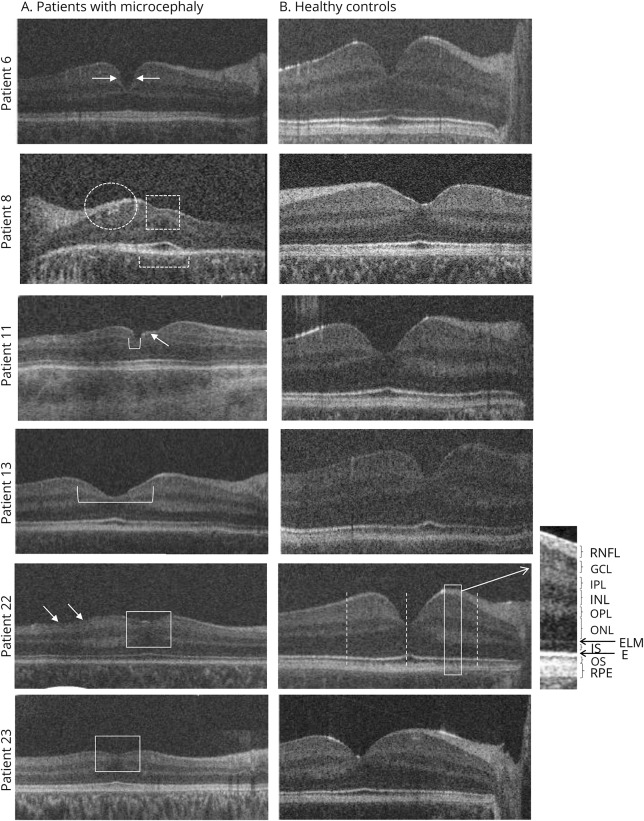
Examples of macular handheld (HH) optical coherence tomography (OCT) of patients with microcephaly vs control participants Patient 6 (de novo *MECP2* duplication) with relatively steep foveal pit contour (arrows). Patient 8 (microcephaly lymphedema chorioretinal dysplasia) has foveal hypoplasia with continuation of the inner retinal layers (open square) and abnormal hyperreflective interfaces between the retinal nerve fiber layer (RNFL) and ganglion cell layer (GCL) (white oval). In addition to disruption of the ellipsoid zone, the outer nuclear layer also appears severely attenuated. The ellipsoid zone is not evident outside the foveola (brackets). Patient 11 has irregular borders (arrow) of a small foveal pit (bracket). Patient 13 displays a broadened foveal pit (bracket). Patients 22 and 23 (possible progressive encephalopathy with edema, hypsarrhythmia, and optic atrophy) with foveal hypoplasia (open squares) due to continuation of the inner retinal layers. Abnormal hyperreflective interfaces between the RNFL and GCL are also evident (arrows).

Macular OCT scans were acquired for all 27 patients (from both eyes in 23 out of 27 patients). The scan with the best image quality was analyzed, including 10 macular OCT scans from the right eye and 17 from the left eye. Initial visual inspection of the horizontal macular OCT scans of patients revealed a thinner parafoveal retina. Another common finding in microcephalic patients was disruption of the photoreceptor EZ (figure 4, patient 8), abnormal hyperreflective interfaces between the RNFL and GCL (figure 2F and figure 4, patients 22 and 23), and an abnormal morphology of the foveal pit (figure 4, patients 6, 11, and 13). Five patients displayed foveal hypoplasia with continuation of the normally absent inner retinal layers (RNFL, GCL, IPL, INL, and OPL) at the fovea (figure 2F, figure 3, I and J, and figure 4, patients 22 and 23). In total, 85% of patients had abnormal retinal structure on HH-OCT (70% had abnormal foveal structure and 15% had abnormal peripheral retina).

##### Parafoveal retinal layer thickness

**Figure 5:**
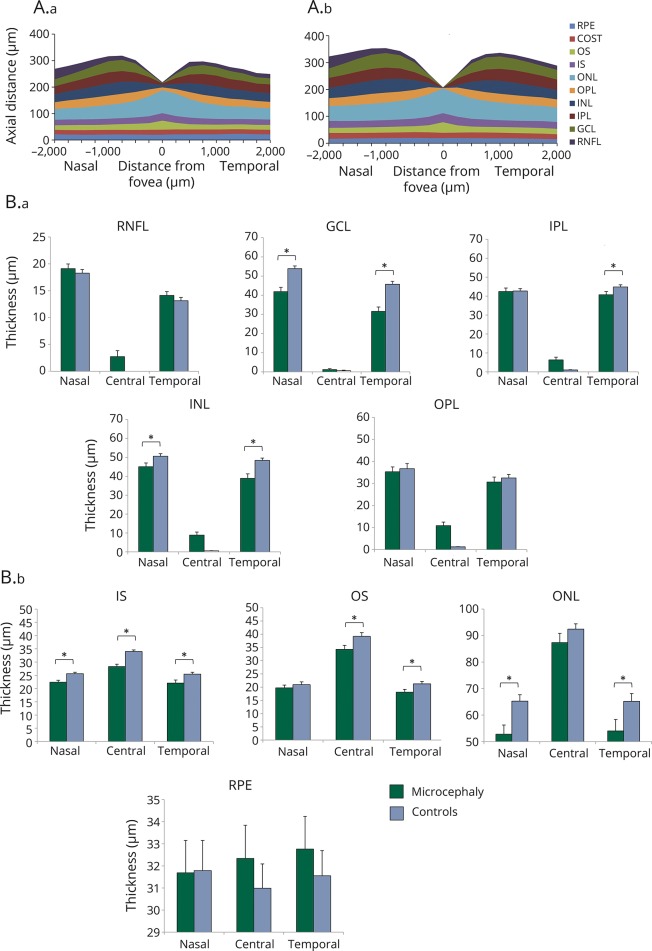
Cross-sectional schematic diagram of individual retinal layers and retinal layer thicknesses (A) Cross-sectional schematic diagram of individual retinal layers in patients with microcephaly (A.a) and in controls (A.b). (B) Mean and standard error (μm) of the thickness of processing (B.a) and photoreceptor (B.b) layers in the center of the fovea and nasally and temporally. GCL = ganglion cell layer; INL = inner nuclear layer; IPL = inner plexiform layer; IS = inner segment; ONL = outer nuclear layer; OPL = outer plexiform layer; OS = outer segment; RNFL = retinal nerve fiber layer; RPE = retinal pigment epithelium. *Significant difference between groups, *p* ≤ 0.05.

Quantitative analysis of OCT scans showed that patients had significantly thinner retinae compared with controls, both nasally (mean ± standard error of the mean: 307 ± 8.5 vs 342 ± 3.7 μm, *p* < 0.001) and temporally (279 ± 11.5 vs 325 ± 3.1 μm, *p* < 0.001). Patients with microcephaly had thinner GCL, INL, ONL, and IS on nasal, temporal, or both sides of the fovea compared to healthy participants (figure 5A). Reduced nasal retinal thickness was caused by thinning of the GCL (42 ± 2.2 vs 54 ± 1.4 μm, *p* < 0.001), INL (45 ± 1.9 vs 50 ± 1.4 μm, *p* = 0.029), ONL (53 ± 3.5 vs 65 ± 2.4 μm, *p* = 0.005), and IS (22 ± 0.7 vs 26 ± 0.5 μm, *p* = 0.001). Reduced temporal retinal thickness was also due to thinning of the GCL (32 ± 2.3 vs 46 ± 1.5 μm, *p* < 0.001), INL (39 ± 2.4 vs 48 ± 1.2 μm, *p* = 0.001), ONL (54 ± 4.3 vs 65 ± 2.9 μm, *p* = 0.023), and IS (22 ± 1.1 vs 25 ± 0.7 μm, *p* = 0.014). In addition, patients with microcephaly had thinner IPL temporally (41 ± 1.8 vs 45 ± 1.1 μm, *p* = 0.047) and thinner OS temporally (18 ± 1.1 vs 21 ± 0.9 μm, *p* = 0.014) compared to healthy participants (figure 5B).

##### Foveal retinal layer thickness

At the foveal center, on average total retinal thickness of patients with microcephaly was similar between both groups, due to a combination of foveal hypoplasia (continuation of the inner retinal layers—RNFL and INL—causing a thicker retina) in 5 patients and thinning of outer retinal layers (figure 5A). Patients with microcephaly had significantly thinner IS (28 ± 0.9 vs 34 ± 0.6 μm, *p* < 0.001) and OS layers (34 ± 1.5 vs 39 ± 1.4 μm, *p* = 0.042). Age and spherical equivalent, which were included as covariates in the statistical model, were not significant factors accounting for the differences found between microcephaly and normal participants.

#### Optic nerve head morphology

**Figure 6:**
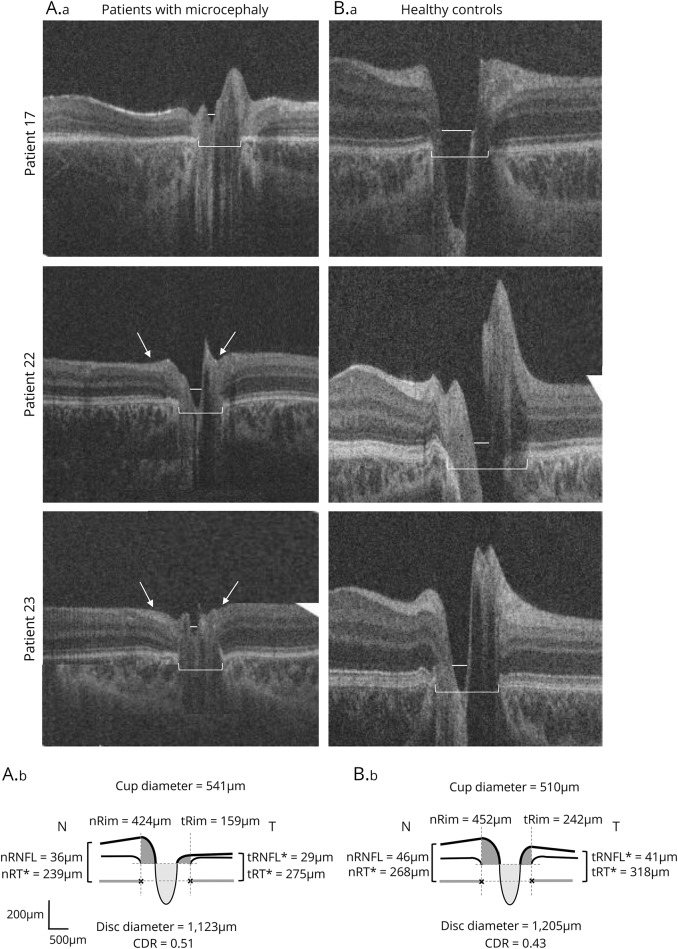
Examples of optic nerve handheld optical coherence tomography of patients with microcephaly vs control participants Patient 17 (microcephaly lymphedema chorioretinal dysplasia) with smaller disc (bracket) and cup (line) diameter. Patient 22 (possible progressive encephalopathy with edema, hypsarrhythmia, and optic atrophy [PEHO]) with reduced peripapillary retinal nerve fiber layer (RNFL) thickness (arrows) and smaller disc (bracket) and cup (line) diameter. Patient 23 (possible PEHO) with reduced peripapillary RNFL thickness (arrows) and smaller disc (bracket) and cup (line) diameter. (Lower) Cross-sectional schematic diagrams represent mean values of optic nerve head measures of patients with microcephaly (left) and controls (right). Upper horizontal dotted lines represent horizontal offset (150 μm) used to determine cup diameters, and lower horizontal dotted lines indicate disc horizontal diameters. Vertical dotted lines show margins of rim areas. Significant values are marked with an asterisk. CDR = cup-to-disc ratio; n = nasal; t = temporal.

Optic nerve head scans were available for 22 out of 27 patients (from both eyes in 19 out of 22 patients). It was not possible to obtain reliable optic nerve head scans in 4 patients (patients 1, 11, 18, and 19) due to motion artifacts. Patient 8 was not included in the optic nerve analysis due to the presence of a falciform retinal fold distorting the optic disc. Analysis included 8 optic nerve head scans from the right eye and 14 from the left eye. Sizes of optic discs and cups were variable. Figure 6 shows examples of small optic nerves in 3 patients with small disc and cup size.

Quantitative OCT analysis revealed that average cup diameter, cup depth, and rim diameter were not significantly different between patients and healthy controls (figure 6, lower panel). Patients with microcephaly had smaller disc diameters (1,123 μm) and larger cup-to-disc ratio (CDR) (0.51) compared to healthy controls (disc diameter 1,205 μm and CDR 0.43), but those differences did not reach statistical significance. Peripapillary RNFL was significantly thinner at the temporal side of the disc in patients with microcephaly compared to controls (29 vs 42 μm, *p* < 0.001). Total peripapillary retinal thickness was smaller in patients with microcephaly compared to controls for both temporal (275 vs 318 μm, *p* < 0.001) and nasal sides (239 vs 268 μm, *p* = 0.013). Age and spherical equivalent, which were included as covariates in the statistical model, were not significant factors accounting for the differences between microcephaly and normal participants.

When converting the visual acuity of fix and follow to 2.28 logMAR visual acuity,^20^ we did not find a correlation between visual acuity and GCL or peripapillary RNFL thickness. However, it is unlikely that the visual acuity of fix and follow obtained in 7 out of our 27 patients corresponded to the visual acuity of hand motion, as many patients were not cooperative and probably had better visual acuity in reality.

#### Neuroimaging findings

Neuroimaging studies (MRI or CT) were available for 22 out of 27 patients. Findings included generalized cerebral atrophy (patients 6, 17, 22, and 23), plagiocephaly (figure 3, G and H, patients 6, 14, 22, and 23), atrophy or agenesis of the corpus callosum (patients 5, 6, and 15), mild periventricular leukomalacia (patient 7), a pituitary microadenoma (patient 7), delay of myelination in the internal capsules, and a mega cisterna magna (figure 3, A and B, patient 27).

## Discussion

This report describes neuro-ophthalmologic and ocular features of a large group of patients with microcephaly and provides quantitative data for structural retinal and optic nerve changes. HH-OCT has enabled the examination of patients who cannot cooperate with conventional table-mounted instruments, showing that microcephaly is almost always accompanied by retinal abnormalities that are often subclinical but that can easily be detected by HH-OCT. Patients with microcephaly displayed abnormal foveal morphology, disruption of the EZ, and a thinner parafoveal retina due to reduced thickness of the GCL, INL, and ONL. In total, 85% of patients with microcephaly had retinal changes on OCT, vs only 33% on funduscopy. The peripapillary retinal and RNFL thickness was also reduced in patients with microcephaly as compared to normal participants.

Hypermetropic astigmatism was the most common refractive error, in accordance with previous case reports and small case series.^8^ In recent studies, the majority of patients with autosomal recessive microcephaly with chorioretinopathy had hypermetropic astigmatism.^5,21,22^ Patients with nanophthalmos and posterior microphthalmos can show macular folds and foveal hypoplasia with absence of foveal depression due to continuation of all retinal layers throughout the macular area.^23,24^ In our study, 2 siblings with MLCRD (patients 17 and 18) with high hypermetropia had foveal hypoplasia with mild macular elevation and low visual acuity, without macular folds. The underlying mechanism for hypermetropia may be a reduction in eye size parallel to the reduction in head circumference observed in microcephaly, as it has been shown in knockdown zebrafish models.^11^ However, in the present study, only 5 out of 27 patients with microcephaly had hypermetropia above +4 spherical equivalent. As there was selective thinning in some but not all of the retinal layers on OCT, microphthalmia with overall reduced ocular size is unlikely to account for the retinal changes we observed in microcephaly. In addition, age and spherical equivalent, which were included as covariates in the statistical model, were not significant factors accounting for the differences found between patients with microcephaly and normal participants.

Nystagmus, previously associated with microcephaly, was a common feature in our patients.^21,22,25,26^ All affected individuals of both families with *KIF11*-negative MLCRD had nystagmus.

Retinal changes in several of our patients were similar to a recent study in microcephaly due to presumed intrauterine Zika virus infection, where the most commonly detected changes were focal pigment mottling of the retina and chorioretinal atrophy, followed by optic disc hypoplasia and severe cupping.^9^ In patients with microcephaly, HH-OCT helped to detect retinal abnormalities, which were not evident on fundus examination. Therefore, it is likely that OCT examination could help with early detection, identification, and quantification of retinal and optic nerve changes that are not apparent on clinical examination in microcephaly, including infectious forms, for example caused by the Zika virus.^9^

Overall, patients displayed a thinner parafoveal retina mainly due to reduced thickness of the GCL, INL, and ONL, and discontinuation of EZ. Interestingly, our study shares several similarities with a recent report of 8 infants with Zika infection showing retinal thinning, discontinuation of the EZ, and hyperreflectivity underlying the RPE (figure 2F).^13^ The present findings are also in line with a recent OCT study reporting atrophic maculae and parafoveal discontinuation of the EZ and the outer retinal bands in patients with pathologic *KIF11* mutations.^5,27^ In this study, macular atrophy was reported for the first time in 3 out of 4 individuals with microcephaly with or without chorioretinopathy, lymphedema, or intellectual disability and is most probably associated with the identification of *KIF11* mutations in all affected patients.^5^ It appears that the variety of ocular findings in this syndrome represent variable expression of a single genetic entity, attributed to the *KIF11* mutations.^21^

Five of our patients had foveal hypoplasia with continuation of the inner retinal layers and thinning of the IS at the fovea, suggesting arrested foveal development with reduced outward migration of inner retinal layers in microcephaly. Foveal hypoplasia in association with microcephaly has been reported previously in a male infant with Amish microcephaly,^28^ and in 2 siblings with a lethal form of microcephaly and primordial dwarfism.^29^ However, these observations were based on funduscopy only. Foveal hypoplasia has been previously reported mainly in ocular albinism, PAX6 mutations, achromatopsia, and prematurity.^30–32^ Recently, foveal hypoplasia has been found to be strongly associated with optic nerve hypoplasia and septo-optic dysplasia, suggesting an association between the development of the optic nerve and fovea.^18^ Unique foveal structures with abnormal contour of the fovea and abnormal hyperreflective interfaces between the RNFL and GCL on OCT has been noted in the present study (figure 3). In addition to foveal hypoplasia, there were severe abnormalities of foveal configuration. In some patients, the foveal pit was steeper; in others, it was wider and shallower, and one patient showed an irregular contour of the foveal pit (figure 4). We have also found disruption of the EZ at the foveal center and parafoveal disruption of the EZ. Similarly it has been found that morphant and mutant zebrafish embryos typically lacked the normal retinal lamination patterns apparent in wild-type embryos, suggesting possible delayed development.^11^

Optic disc hypoplasia has been reported previously in one individual with autosomal recessive microcephaly due to *TUBGCP4* mutations.^22^ Similarly, 4 patients in the present study were funduscopically diagnosed with optic disc hypoplasia confirmed by HH-OCT. Quantitative ONH analysis on HH-OCT also showed that patients with microcephaly had smaller disc diameters and larger CDR, but those differences did not reach statistical significance, possibly due to large variability between patients. This is similar to the increased disc cupping of Zika-infected infants.^9^ HH-OCT additionally showed significantly reduced peripapillary retinal and RNFL thickness in microcephaly, likely related to the reduced size of the corresponding retinal ganglion cell layer found in the central retina. This is in accordance with macular OCT changes in glaucomatous eyes suggesting a relation between the thinning of the macular RGC layer and that of the RNFL at the disc.^33–35^

In microcephaly, the thinning of several retinal layers and the decreased optic disc diameter and rim is likely due to a reduction in retinal cell number and retinal size, parallel to the reduction in brain neurons and brain size.^36,37^ Specifically in patients with MLCRD, recent OCT findings have suggested a severe reduction of the retinal ganglion cell population or their axons, as well as an inappropriate laminar structuring of the different retinal cell populations.^36^ In addition, it has been hypothesized that severely disorganized retinae of mutant fish embryos with patchy areas of increased fluorescence are suggestive of cell debris.^11^ Brain size during birth and the ultimate number of neurons are determined by the balance between cell proliferation and cell death during neurogenesis. It has been suggested that human primary microcephaly is a disorder of neurogenic mitosis occurring in the brain, optic nerve, and retina.^38–40^ Other theories point to a primary inability to emit axons, and impaired development or closure of the optic nerve fissure with subsequent alteration of the retinal ganglion cells.^41,42^ It is not clear why significant thinning was found in only specific retinal layers in patients with microcephaly: the photoreceptor layers including the IS, the ONL, the INL, the GCL, and the peripapillary RNFL. Possibly photoreceptors and specifically the EZ are more vulnerable to arrested mitosis due to higher energy demands.

Expression of genes associated with microcephaly occurs in the brain, retina, and optic nerve. This is in agreement with retinal and optic nerve changes in microcephaly. For example, the *KIF11* gene, which likely plays a role in retinal vascular development,^43^ is strongly expressed in proliferating embryonic tissues including the brain, the spinal cord, and a proliferative region at the periphery of the retina.^44^ Furthermore, additional genes associated with microcephaly such as the *COH1* gene, the *NBS1* gene, and the *CDK19* gene are all expressed in the human brain and retina.^45–47^

A limitation of our study is that the underlying etiology and genetic abnormalities were heterogeneous, and partially the etiology of the microcephaly was unknown. Four out of 8 patients with microcephaly with seizures were treated with vigabatrin and median duration of vigabatrin use was 19.5 months (range 7–24). In some patients, vigabatrin is known to cause retinal toxicity, resulting primarily in concentric peripheral visual field loss without changes of the retina or optic nerve on funduscopy.^48^ Visual field testing was not possible in our patients taking vigabatrin due to reduced cooperation. Recent studies in children and adolescents indicate that vigabatrin is associated with peripapillary RNFL thinning.^49,50^ Therefore, we cannot exclude that vigabatrin has contributed to the reduction of the peripapillary RNFL layer in our study. However, our data suggest specific retinal and optic nerve findings in microcephaly of various etiologies. Furthermore, the present OCT data were acquired using a HH-OCT device. The learning curve for this technology is approximately 2 weeks, and after this reasonable training period HH-OCT becomes user-friendly and is noninvasive and fast. The high success rate of obtaining scans has currently established its use for almost all pediatric patients in our department.

The present series adds significantly to our knowledge of the retinal and optic nerve findings in patients with microcephaly, possibly enabling more accurate diagnosis and management of patients with these rare disorders by detailed analysis of the retina and optic nerve using OCT. We showed that patients with microcephaly have selective thinning in several retinal layers, mainly in the IS and the GCL, and also demonstrated a reduction in peripapillary nerve fiber layer and peripapillary retinal thickness. Severe foveal abnormalities were also found. The developmental anomalies in the retina and optic disc are likely to be caused by impaired neurogenic mitosis in microcephaly, and reflect a reduction in retinal cell population and alterations in normal retinal lamination patterns. Our study provides evidence that microcephaly is almost always associated with retinal and optic nerve abnormalities, which are often not apparent on clinical examination, but can be easily detected and quantified by HH-OCT. Therefore it is advisable to obtain HH-OCT in children with microcephaly. The diagnostic potential of HH-OCT will enable the early detection of retinal lesions and will possibly improve phenotyping and differentiating subtypes of microcephaly.

## Glossary

CDR: cup-to-disc ratio
EZ: ellipsoid zone
GCL: ganglion cell layer
HH: handheld
ICC: intraclass correlation coefficient
INL: inner nuclear layer
IPL: inner plexiform layer
IS: inner segments of the photoreceptors
MICPCH: microcephaly with pontine and cerebellar hypoplasia
MLCRD: microcephaly lymphedema chorioretinal dysplasia
OCT: optical coherence tomography
ONH: optic nerve head
ONL: outer nuclear layer
OPL: outer plexiform layer
OS: outer segments of the photoreceptors
PEHO: progressive encephalopathy with edema, hypsarrhythmia, and optic atrophy
RNFL: retinal nerve fiber layer
RPE: retinal pigment epithelium
